# Technology-Based Interventions in Tobacco Use Treatment Among People Who Identify as African American/Black, Hispanic/Latina/o, and American Indian/Alaska Native: Scoping Review

**DOI:** 10.2196/50748

**Published:** 2024-10-10

**Authors:** Emily Hichborn, Avery Turner, Sarah Moore, Phoebe Gauthier, Kathleen Bell, LaTrice Montgomery, Jesse Boggis, Chantal Lambert-Harris, Elizabeth Saunders, Jesse Dallery, Bethany McLeman, Lisa Marsch

**Affiliations:** 1 Center for Technology and Behavioral Health Geisel School of Medicine Dartmouth College Lebanon, NH United States; 2 College of Medicine University of Cincinnati Cincinnati, OH United States; 3 The Dartmouth Institute for Health Policy and Clinical Practice Geisel School of Medicine Dartmouth College Hanover, NH United States; 4 College of Liberal Arts and Sciences University of Florida Gainesville, FL United States

**Keywords:** health disparities, underrepresented, social determinants of health, tobacco use, technology-based interventions, scoping review, mobile phone

## Abstract

**Background:**

Although tobacco use has significantly declined in the general population, traditional tobacco use treatment uptake and success rates remain disproportionately low among people who identify as African American/Black, Hispanic/Latina/o, and American Indian/Alaska Native. Technology-based interventions (TBIs) for tobacco use are promising alternatives to traditional tobacco use treatments.

**Objective:**

This scoping review aims to investigate the extent to which the use of digital TBIs in tobacco use treatment research promotes health equity among people who identify as African American/Black, Hispanic/Latina/o, and American Indian/Alaska Native.

**Methods:**

This scoping review identifies US-based studies (between January 2000 and March 2021) that enlist TBIs for tobacco use treatment and include people who identify as African American/Black, Hispanic/Latina/o, and American Indian/Alaska Native at ≥50% of the sample when combined; features studies that are also race and ethnicity conscious; and highlights health equity–promoting insights from included studies.

**Results:**

In 85% (22/26) of the studies, the largest proportion of the sample was African American/Black, most participants had low socioeconomic status, and recruitment was most commonly from medical settings. In total, 58% (15/26) of the studies were race and ethnicity conscious, and 67% (10/15) of these studies sought to partner with potential end users. An array of TBIs were represented; however, SMS text messaging was most prevalent. Most TBIs were combined with other evidence-based intervention components (eg, nicotine replacement therapy). Approximately one-third of the studies (8/26, 31%) required participants to have their own device or internet access. The majority were underpowered to detect substantial differences.

**Conclusions:**

The modest number of studies, particularly for persons who identify as Hispanic/Latina/o and American Indian/Alaska Native, demonstrates the limited application of TBIs for tobacco use and that additional research is needed to determine the extent to which TBIs for tobacco use promote health equity among these populations.

**International Registered Report Identifier (IRRID):**

RR2-10.2196/34508

## Introduction

### Background

Pharmacological and behavioral treatments have demonstrated success in reducing tobacco use in the general population, although the use of these services remains disproportionately low among people who identify as African American/Black, Hispanic/Latina/o, and American Indian/Alaska Native [1]. People who use tobacco and identify as African American/Black and Hispanic/Latina/o are more likely to have made a quit attempt in the past year than White people who smoke [2]; however, their attempts to quit are less likely to be successful due, in part, to their lower use of nicotine replacement therapy (NRT) and other cessation services [2,3]. Hispanic/Latina/o and American Indian/Alaska Native people who smoke are less likely to receive advice to quit from a health professional than White people who smoke [2]. People who identify as American Indian/Alaska Native have the highest smoking prevalence of any racial or ethnic group in the United States [4]. The lack of antitobacco laws on tribal lands and the predatory advertising practices used by tobacco companies to target individuals who identify as American Indian/Alaska Native contribute to the high rates of tobacco use in this group [5]. While effective pharmacological and behavioral treatments for tobacco use exist, higher smoking rates and lower quit success rates among people who identify as African American/Black, Hispanic/Latina/o, and American Indian/Alaska Native indicate that traditional approaches to tobacco use have not yielded optimized and proportional benefits to these populations.

This confluence of factors suggests that novel approaches are needed to address tobacco use among members of racial and ethnic underrepresented groups [6]. Technology-based interventions (TBIs) include the use of technologies, such as computer-based and web-based interventions, SMS text messaging (TM), interactive voice recognition, smartphone apps, and other emerging technologies, to deliver interventions aimed at reducing tobacco use [6,7]. According to the National Telecommunications and Information Administration, 76.9% of Americans who identify as African American/Black, 76.7% of Americans who identify as Hispanic/Latina/o, and 74.6% of Americans who identify as American Indian/Alaska Native reported accessing the internet at least occasionally using any type of device in the past 6 months [8]. Because the National Telecommunications and Information Administration began collecting this information in 1994, internet use has dramatically increased across all racial groups in the United States [8]. Disparities persist in internet access among persons with disabilities, older persons, Americans with low socioeconomic status (SES), and Americans who identify as African American/Black and Hispanic/Latina/o; however, these groups increased their internet use between 2019 and 2021, while use remained stagnant for Americans who identify as White in the same time period [9].

Owing to widespread access to technology in the United States, TBIs are a promising approach to tobacco use treatment and provide an opportunity to promote health equity among marginalized and underserved communities [10]. TBIs increase reach to vulnerable populations by decreasing informational barriers and allowing patients on-demand access to innovative, cost-effective tobacco use treatments [11] without the traditional barriers to accessing treatment, including high costs of treatment and travel to clinics and low rates of treatment fidelity [12]. In addition, some TBIs allow clinicians and researchers greater access to real-time patient data, allowing them to provide more personalized tobacco use treatment [11] and the opportunity to prevent, detect, and resolve health disparities in a timelier manner [10]. The development and implementation of effective TBIs for positive health behavior change should result in decreased use and cost of health services over time [13]. Owing to the high prevalence of internet use among people who identify as racial or ethnic minority individuals and the increased access to tobacco use treatment provided by TBIs, TBIs are a promising approach to address tobacco use among members of underrepresented populations [11].

In addition to providing increased access to treatment, TBIs for tobacco use have demonstrated effectiveness [14]. Two recent Cochrane reviews on the use of mobile phones [15] and internet-based interventions [16] for tobacco use support both the prevalence and promise of TBIs. Whittaker et al [15] analyzed 12 studies that used mobile phone–based tobacco use treatment and found that smokers who were given access to mobile phone–based interventions were 1.7 times more likely to have remained abstinent 6 months after the treatment than smokers who did not have access to the TBI. Taylor et al [16] reviewed 67 randomized controlled trials (RCTs) that used internet interventions for tobacco use. They found that tailored and interactive internet-based tobacco use treatment increased smoking cessation at 6 months after the treatment when compared with nonactive controls, although they cautioned the interpretation of these findings due to high statistical heterogeneity [16]. In addition, they found that internet-based interventions combined with behavioral support were more effective than nonactive controls at increasing cessation rates 6 months after the treatment [16].

While TBIs pose an opportunity to promote health equity among people who identify as African American/Black, Hispanic/Latina/o, and American Indian/Alaska Native, these communities have not historically been included in digital health research [11]. People who identify as African American/Black and American Indian/Alaska Native have traditionally been exploited and traumatized through participation in research, resulting in a deep-seated mistrust in researchers [17,18]. It is imperative that researchers repair relationships with these individuals, as their input is critical for the development and dissemination of equitable and effective TBIs. Digital technologies that are not designed for, or tested with, samples that include members of underrepresented groups may inadvertently exacerbate existing health inequities [11]. Brewer et al [10] highlight the lack of socioculturally tailored TBIs that include people from diverse racial and ethnic backgrounds and warn of the downstream effects of using TBIs that are “acontextually” developed. Without engagement and input from underrepresented communities, TBIs may largely benefit 1 subgroup of the population while perpetuating or even worsening health-related outcomes for another [10]. For example, an analysis of SmokefreeTXT (developed by the National Cancer Institute), a national text-based tobacco use treatment, found that 12.9% of smokers who completed the program self-reported abstinence from tobacco use at the end of treatment [19]. However, African American/Black users of SmokefreeTXT were less likely than White users to report tobacco use abstinence at all time points during treatment [20]. While this study did not meet the criteria for our scoping review, findings such as these underscore the need to ensure that TBIs for tobacco use are being developed, tested, and disseminated equitably. Therefore, an exploration of the research on TBIs for tobacco use that includes people who identify as African American/Black, Hispanic/Latina/o, and American Indian/Alaska Native is warranted.

### Study Aims

The following research question guided this scoping review: To what extent does the use of digital TBIs in tobacco use treatment research promote health equity among people who identify as African American/Black, Hispanic/Latina/o, and American Indian/Alaska Native?

We operationally defined promoting health equity as the *inclusion* of individuals (≥50% of the sample) who identify as African American/Black, Hispanic/Latina/o, and American Indian/Alaska Native in the research of TBIs for tobacco use, as well as the extent to which the research is *race/ethnicity conscious*. To explore whether this subset of research is race and ethnicity conscious, we determined whether the research was explicit about members of underrepresented groups, beyond their inclusion as study participants, in the title, introduction, methods, results, and discussion of the manuscript. We examined peer-reviewed literature published between January 2000 and March 2021 that included individuals who identify as African American/Black, Hispanic/Latina/o, and American Indian/Alaska Native in tobacco use treatment research using TBIs. Given a lack of synthesized findings on the use of TBIs to address tobacco use among underrepresented populations, a scoping review is an ideal tool to provide an overview of TBI studies for tobacco use and explore whether TBIs included in the review promote health equity in tobacco use treatment among underrepresented groups. No current or underway scoping reviews or systematic reviews were identified in the preliminary search.

## Methods

### Original Scoping Review

Studies were initially identified as part of a larger scoping review on TBIs being used in substance use treatment with a majority sample of individuals who identify as African American/Black, Hispanic/Latina/o, American Indian/Alaska Native [21]. This review was conducted using the methodological framework proposed by Arksey and O’Malley for scoping reviews [22], and the results were reported according to the PRISMA-ScR (Preferred Reporting Items For Systematic Reviews And Meta-Analyses Extension for Scoping Reviews) guidelines. Hichborn et al [21] provide additional details on the protocol that covers this tobacco review and the larger scoping review.

### Data Sources

Preliminary literature searches were completed in Google Scholar and PubMed, netting dozens of US-based peer-reviewed research studies that met the scoping review criteria. On the basis of the initial search, 5 electronic databases (MEDLINE, Scopus, Cochrane Library, CINAHL, and PsychINFO) were selected to identify tobacco use treatment research using TBIs (Multimedia Appendix 1). The literature searches were conducted by 2 Dartmouth College reference librarians.

### Study Selection and Inclusion and Exclusion Criteria

Studies eligible for inclusion were peer-reviewed, qualitative, quantitative, and mixed methods studies; randomized trials; RCTs (efficacy and effectiveness); feasibility and acceptability pilots; formative development studies; secondary analyses (eg, mechanisms or moderators); and assessments. Studies were not included if they were considered protocol papers, papers discussing planned or future work, reviews, commentaries, editorials, opinion pieces, student theses, conference abstracts, book chapters, and guidelines. Eligible studies were US-based due to the context-specific and country-specific issues facing the individuals who are the focus of this review. Eligible studies were also conducted in the English language owing to the language fluencies of the study team. Owing to a clear and emerging increase after 2001 surrounding the number of studies published on the design and development of technology-based behavior change interventions [13], eligible studies were selected from those published between January 2000 and March 2021. In addition, eligible studies included a TBI designed for tobacco use treatment with individuals aged ≥12 years and had at least 50% of participants identifying as African American/Black, Hispanic/Latina/o, or American Indian/Alaska Native when combined [23]. Furthermore, studies solely focused on mental health, pharmacological interventions, cost evaluations, telephone counseling only, primary prevention interventions, and substances other than tobacco were excluded.

### Screening and Selection Procedure

Following the initial literature search, all netted studies were uploaded to Endnote X9 (Clarivate Analytics) to remove duplicates [24] and were then transferred to Rayyan (Rayyan Systems, Inc), a web-based tool used to assist researchers in screening, selecting, and labeling studies for systematic reviews [25]. Three teams consisting of ≥2 independent reviewers blinded to each other’s selections first screened all identified studies for inclusion by reviewing the title and abstract (N=6897). Articles meeting the initial criteria were then screened at the full-text level (1159/6897, 16.8%). Through this process, the team identified 21 studies meeting inclusion criteria at both the title and abstract and full-text phases of review. A total of 5 additional studies were identified through background searches, for a total of 26 included studies. Across the selection procedure, all disagreements were addressed through discussion or with additional reviewers.

### Data Extraction

Using a standardized extraction form (Multimedia Appendix 2) with prespecified extraction fields (eg, aims, study design, and year of publication), data were extracted from included studies by members of the scoping review research team. In addition, the inclusion of members of the specific racial and ethnic groups, nature of the TBIs, primary outcomes, and verbatim race- and ethnicity-conscious text segments related to the scoping review research questions were extracted. Notably, while scoping reviews do not typically assess the quality of included studies, we have included a summary of study outcomes to inform readers about the consistency of this subset of the literature with the larger body of research in terms of primary and secondary outcomes and to point readers to studies with statistically significant findings, falling far short of a systematic evaluation of the rigor of the analyses. Information gathered from each included study was documented on the extraction form using Excel (Microsoft Corporation). We piloted our data extraction spreadsheet with 2 studies to ensure consistency and allow for iterative revision of the extraction variable definitions before the extraction was completed. Quality assurance checks were completed for 100% (N=26) of the sample by a second team member who reviewed the extractions to ensure accuracy, consistency, and thoroughness, primarily focusing on fields with the potential for greater variability. One author was contacted for additional clarification on his program of research.

### Race and Ethnicity Consciousness

All included studies were evaluated for evidence of race and ethnicity consciousness in the development, conduct, analysis, and presentation of research findings. Studies were considered race or ethnicity conscious if the published manuscript included consideration of or was explicit about at least one of the aforementioned underrepresented racial or ethnic groups in one or more manuscript sections (ie, title, introduction, method, results, and discussion; Textbox 1).

**Examples of race- or ethnicity-conscious practices in research.**

**Title**

Signaling the focus on one or more racial or ethnic groups in study titles, for example, “‘Every day I think about your messages’: assessing text messaging engagement among Latino smokers in a mobile cessation program” [26].

**Introduction**

Provide epidemiological or other relevant information about one or more racial or ethnic groups in the literature review or enlist a theory that is described as one that may help address health disparities among underrepresented groups. An example of this includes sociological trust theory—a bridge between a broad lens of culturally informed design and attention to trust or distrust.

**Methods**

Race- and ethnicity-conscious methods may include references to recruitment or retention efforts aimed at racial and ethnic groups, such as cultural tailoring of materials or consideration of matching staff race or ethnicity to that of the sample participants (in telemedicine appointments, and in the animations seen in virtual reality, and in computer games), and assessment for measurement equivalence. Race- or ethnicity-conscious analytic plans may include conducting separate analyses for each race and ethnicity, focusing on within-group differences rather than race comparisons, using stratification methods that balance each race and ethnicity across treatment arms, or in some other way considering race or ethnicity in the plan for analyzing the data.

**Results**

Present findings in ways that highlight differences and similarities for members of different racial or ethnic groups.

**Discussion**

Interprets findings for members of racial or ethnic groups by locating results in the context of other development or treatment literature.

### Data Synthesis and Analysis

On the basis of the guiding research questions regarding inclusion and race and ethnicity consciousness, tables were populated with data extracted relevant to the research questions (ie, refer to Multimedia Appendices 3 [26-51] and 4 [26-51]). The remaining data extracted served to populate additional tables that helped to describe the studies (Multimedia Appendix 5 [26-51]) as well as the range and nature of the TBIs (Multimedia Appendix 6 [26-51]).

## Results

### Descriptions of Included Studies

**Figure 1:**
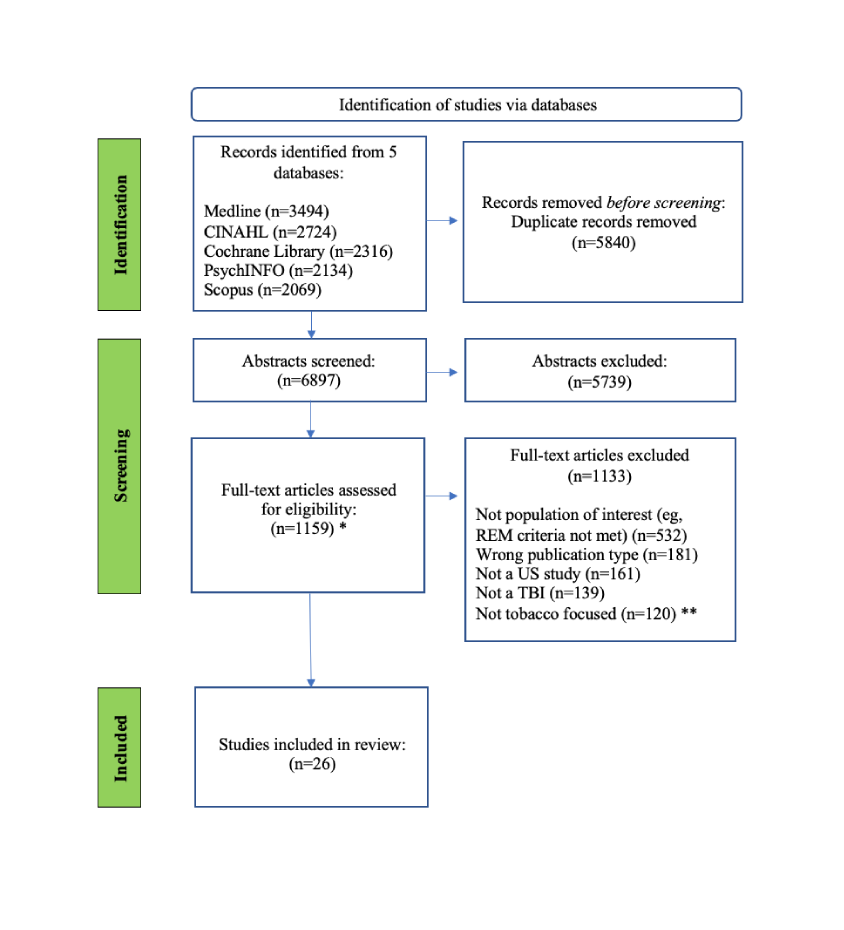
Identification of studies. TBI: technology-based intervention. *One study was identified during a review of references after the initial search but was not ultimately included in this review. **Includes studies that focused on substances other than tobacco reviewed separately (see [21]). Tobacco use treatment literature was separated from the substance use treatment literature because until recently, many clinicians and scientists regarded tobacco use disorder and substance use disorder as separate. Substance use clinicians face barriers such as limited training on tobacco use treatment and limited resources for addressing tobacco use in substance use treatment settings, resulting in a focus on attaining abstinence from all substances other than tobacco [23, 24]. Clinicians and scientists have advocated for the inclusion of tobacco use treatment in substance use treatment settings, however, given the distinct way in which tobacco use is treated in systems of care compared to other substance use, we examined the tobacco use studies separately.

Studies that met eligibility criteria were published between January 2000 and March 2021 and represent a range of research designs: RCTs (12/26, 46%) [27-38]; randomized trials (4/26, 15%) [39-42]; formative and development studies (4/26, 15%) [30,34,36,43], including Mason et al [30] and Orr et al [34], which are also counted among RCTs as publications present findings for both designs; feasibility, acceptability, and effectiveness studies (3/26, 12%) [26,37,44]; secondary data analyses (3/26, 12%) [45-47]; and qualitative analysis (1/26, 4%) [48]. The TBIs were most often combined with other evidence-based intervention components: NRT (11/26, 42%) [27-29,32-34,36,40-43], pre- or postcessation counseling (9/21, 35%) [27,28,32-34,36,41,43,44], printed informational manual, brochure, or reports (5/26, 19%) [28,29,35,36,40], and contingency management (3/26, 12%) [27,39,43]. About 27% (7/26) were stand-alone interventions [26,30,37,38,49-51]. Figure 1 [52-54] shows an overview of the identification of studies.

### Descriptions of TBIs for Tobacco Use

Multimedia Appendix 6 presents detailed information, including the location of TBI delivery. The range of digital tools supporting the TBIs included apps accessed via the internet (3/26, 12%) [27,43,44], computer-delivered programs (3/26, 12%) [28,29,39], SMS TMs (16/26, 62%) [26,30-37,40,45-49,55], virtual reality (VR; 2/26, 8%) [42,50], DVDs (1/26, 4%) [51], and PDAs (1/26, 4%) [38]. The dosage of exposure and intervention intensity varied widely: apps ranged from 2 times daily for 30 days [44] to 2 times daily for 7 weeks [27,43]; computer-delivered programs ranged from 1 [39] to 8 weekly sessions [28]; SMS TM interventions ranged from 6 to 9 TMs over 5 days [30] to 4 TMs per day over 18 months [41]; VR programs ranged from 7 [50] to 10 [42] weeks of 45 [50] to 60 [42] minute sessions; the lone DVD program exposed participants to two 60-minute videos with 1-month latency between views [51], and the lone PDA-delivered intervention exposed participants to one 20-minute meditation per day for 2 weeks [38]. Of 26 studies, 10 (38%) study teams sought to partner with potential end users, engaging them in the development or adaptation of a TBI [28,34,36,37,39,43,44,48-50] and 12 (46%) study teams did not [27,29,30,32,33,35,38,40-42]. The remaining 4 (15%) studies were secondary analyses by Mason et al [30] and therefore not double counted here. Finally, almost all included studies can be grouped by racial or ethnic cultural focus: 23% (6/26) African American/Black [30,31,45-47,51], 8% (2/26) American Indian/Alaska Native [34,41], 4% (1/26) Hispanic/Latina/o [26] *or* a handful of target populations: 19% (5/26) pregnant and parenting individuals [32,35,37,39,49]; 15% (4/26) people living with comorbidities (ie, HIV [28,36]; schizophrenia, schizoaffective disorder, or psychotic disorder [43]; and posttraumatic stress disorder [27]), 8% (2/26) people experiencing unemployment or economic disadvantage [29,33], and 4% (1/26) adolescents [50]. Of the 26 studies, 5 (19%) [38,40,42,44,48] recruited convenience samples.

### Summary of Study Outcomes

The strictest outcome definition in the field of tobacco use cessation research, bioverified smoking cessation (BVSC), was a main outcome measure for 11 (42%) of the 26 studies [26-29,32,33,35,39,41,42,44]. Of 26 studies, 2 (8%) TBIs, including computer-delivered 5As [39] and tailored telemedicine plus NRT with phone counseling [33], demonstrated statistically significant differences relative to respective comparators (*P*=.02 [39], *P=*.03 [33]). Bioverified smoking reduction was the primary outcome measure for 1 (4%) study [42]. Smoking rates were significantly lower (*P*=.045) for the VR skills training group compared with the NRT-only group at the end of treatment. Self-reported smoking cessation (SRSC) was the other primary outcome measure of note across studies [27,29,33,37,38,40,41,43,44,50,51]. TBIs that demonstrated statistically significant differences in SRSC were SMS TM computer-assisted counselling (*P*=.02) [29]; SmokeFreeTxt (*P*=.003) [40]; tailored TM with NRT, and phone counseling (*P=*.03) [33]; VR TBI; The Breathing Room (*P*=.045) [42]; and Brief mindfulness practice (*P*<.001) [38]. Self-reported smoking reduction was the primary outcome measure for the SMS TM program of research by Mason et al [30,31]. In both reports, statistically significant differences were demonstrated (*P*=.04 [30], *P=*.045 [31]), favoring personalized SMS TM over general health habits SMS TM. It is notable that only 7 (27%) of the 26 studies were sufficiently powered to detect significant findings [28-31,33,35,51]. Multimedia Appendix 5 presents more details, including whether studies were powered to detect differences.

### Tobacco TBI Access and Inclusion: Promoting Health Equity

A total of 13 (50%) of the 26 study samples include at least 75% of one underrepresented group targeted in this review [26,27,30-34,36,39,41-47] (articles by Mason et al counted [51] as 1 study). Of 26 studies, 1 (4%) had 100% Hispanic/Latina/o [26], 1 (4%) had 100% African American/Black [51], 2 (8%) had 100% American Indian/Alaska Native [34,41], and 14 (54%) included at least 75% African American/Black participants [27,30-33,36,39,42-47]. In 22 (85%) of the 26 studies, the largest proportion of the sample was African American/Black. Of 26 studies, 9 (35%) studies included Hispanic/Latina/o participants [26,28,29,33,40,42,48-50]; however, only 2 [26,50] included ≥50% of Hispanic/Latina/o participants. Furthermore, nearly all studies other than those involving adolescents (6/26, 23%) and those that did not describe SES variables (4/26, 15%) reported that most of their participants were either unemployed, had annual incomes below the poverty line, did not have educations beyond the high school level, or had public health insurance or no insurance.

The included studies used a variety of recruitment strategies. The most common of which was recruitment from medical settings (11/26, 42%) [28,32,35,37,39-41,43,44,48,49]. Of the 26 studies, 7 (27%) studies used community-based approaches, such as placing flyers in churches or supermarkets; approaching individuals in heavily concentrated public areas; and placing advertisements on the radio, in newspapers, and on Craigslist [26,33,38,41-43,51]. Two (8%) of the 26 studies relied primarily on snowball or respondent-driven sampling [30,31,45-47,51], 2 (8%) other studies recruited participants primarily from schools or universities [34,50], and 1 (4%) study did not describe its recruitment plan [36].

Retention strategies were defined as any effort to collect follow-up data from participants in studies that involve >1 time point [56]. All included studies except for one [49] attempted to collect follow-up data from participants. Compensation, provision of a device or internet access required to use the TBI (platform availability), and technical support provided were highlighted retention strategies because of their relevance for maintaining engagement in the included studies. Most of the (18/26, 69%) included studies compensated participants [27-31,35-39,41,43-47,50,51], and 2 (8%) of the 26 studies additionally provided travel vouchers [28,36]. Of 26 studies, 8 (31%) studies stipulated that a candidate was eligible only if they owned or had access to a device to engage with the TBIs [26,28,32,36,40,44,48,51]. Several (16/26, 62%) studies did not require participants to have access to, own a device, or have internet service (eg, TBI accessible at the study site) [29,42,50]. Technical support was provided to participants in 13 (50%) of the 26 included studies [27,28,30,31,36-38,43-47,50], facilitating continued engagement with the TBI.

### Race and Ethnicity Consciousness of Included Studies: Promoting Health Equity

Multimedia Appendix 4 provides further details regarding the 26 studies: 4 (15%) studies signaled the focus on an underrepresented group in study titles [26,34,41,51] (eg, A randomized trial to reduce smoking among American Indians in South Dakota: The walking forward study [41]), 12 (46%) explicitly referenced underrepresented groups in the Introduction section [26,30-34,36,41,45,46,51], 11 (42%) exemplified race- or ethnicity-conscious methods [26,30-32,34,39,41,45,46,50,51], 7 (27%) specifically characterized findings among underrepresented groups in the Results section [26,28,32,36,38,39,51], and finally, 11 (42%) addressed a specific underrepresented group in the Discussion or Conclusion sections [26,30,31,34,36,39,41,45-47,51].

## Discussion

### Principal Findings

This scoping review identified 26 studies that were published between January 2000 and March 2021, used a TBI designed for tobacco use treatment with individuals aged ≥12 years, and had at least 50% of participants identifying as African American/Black, Hispanic/Latina/o, or American Indian/Alaska Native when combined. Studies included in this scoping review, by definition, promoted health equity by including a majority of members of underrepresented groups in study samples. Slightly more than half (15/26, 58%) were found to be explicit about the inclusion of these members, as well as the potential impact of the TBIs for members of these groups (ie, race or ethnicity conscious). Although predominantly RCTs, only a handful of included studies (7/26, 27%) were powered to detect significant findings. These studies used a variety of outcomes to assess the effectiveness of TBIs. The range and nature of TBIs included in this review were found to be generally representative of TBIs under investigation for use with people experiencing the full range of substance use disorders (SUDs). The review identified a modest number of studies focused on TBIs for tobacco use treatment that include a majority of people who identify as African American/Black, Hispanic/Latina/o, and American Indian/Alaska Native. In most (22/26, 85%) of the identified studies, participants who identified as African American/Black made up the largest proportion of the sample and most participants reported having low levels of education and income. Participants were recruited from a variety of settings, although most frequently from medical settings, and most (21/26, 81%) of the included studies engaged in efforts to retain participants. Furthermore, multiple study methods’ insights designed to promote health equity warrant attention.

### Inclusion in Research

Despite the disproportionate impact of tobacco use on people who identify as African American/Black, Hispanic/Latina/o, and American Indian/Alaska Native [57-60]; the exponential growth in the development of and research on TBIs for behavioral health [13], SUDs [61,62]; and tobacco use specifically [55,63-65] in the United States since 2000, one key finding of this scoping review is that *a modest number of studies met inclusion criteria* (N=26). In 85% (22/26) of the included studies, the largest proportion of participants identified as African American/Black. Only 4 (15%) of the 26 studies included samples with more than half of the participants identifying as Hispanic/Latina/o or American Indian/Alaska Native. *The dearth of research on TBIs for tobacco use treatment among all racial and ethnic groups is problematic: for people who identify as Hispanic/Latina/o or American Indian/Alaska Native, it is profound.* Recruitment of sufficient numbers of racial and ethnic minority participants is imperative to understanding how research affects underrepresented groups [66]. Underrepresentation in research limits the extent to which members of these groups benefit from advances in tobacco use treatment [67].

The prevalence of low SES among participants in the included studies is also notable. Given the extensively supported association between tobacco use and SES factors, such as education and poverty [68-71], the finding that approximately one-third of the studies (8/26, 31%) required participants to own or have access to a device or internet to engage with the TBIs suggests a recommendation for reducing barriers to access among low SES populations in future studies. Despite often-repeated statistics regarding universal access, Americans with lower incomes, who are disproportionately members of underrepresented racial and ethnic groups, have lower levels of technology adoption [72-74]. This pattern holds for smartphones, desktop or laptop computers, home broadband access, and tablet computers. Although not the responsibility of investigators alone, one way that may lower barriers to study enrollment is to make devices and the internet more accessible to participants. Relatedly, removing the requirement for participants to use a specific technological device and instead allowing participants to use a currently owned or preferred device may help promote health equity. However, it is important to highlight that while providing the technology required may reduce some barriers to engagement, other considerations, such as digital literacy, serious mental illness, and organizational policies, may also have a significant impact on real-world populations and should be considered in both future research and clinical applications [75]. Another practice for including individuals who identify as African American/Black, Hispanic/Latina/o, American Indian/Alaska Native is to use community involvement and outreach methods (including respondent-driven sampling, community-based participatory research, and recruiting at locations within the target group’s community), which have been shown to be more effective than other methods to reduce study enrollment barriers, thus increasing intervention participation [66]. *The most common recruitment strategy among studies included in this review was recruitment from medical settings,* which may favor insured persons and limit access for members of underrepresented groups due to pervasive mistrust of the medical community [76-80], particularly with respect to research [81]. On the basis of our review of the literature, the inclusion of underrepresented groups recruited from a broad array of settings with a diverse array of methods will help ensure increased representation and generalizability. Suggested alternative settings include community treatment programs, centers, and housing; social media; places of worship; and health fairs through community-based participatory research and respondent-driven sampling [66,82,83].

### TBI Findings

The heterogeneity of TBIs for tobacco use included in this review appears to be representative of the larger universe of TBIs for SUDs [7], as does *the high number of TM interventions relative to other platforms* [65]. SMS TM is less technologically complex compared with other digital platforms and being cost-effective, it is also the most popular mobile phone feature nationally among people in SUD treatment, as well as primary care [7,84]. Importantly, 35% of US Latina/o adults and 24% of African American/Black adults rely on smartphones as their primary means of internet access at home [85]. Therefore, mobile health interventions, including SMS TM interventions, may be a first-tier option to address tobacco use among people who identify as Latina/o and possibly also African American/Black if the goal is to lower barriers to access for all [86].

Consistent with the findings of systematic reviews of mobile phone–based [15] and web-based interventions [16] for tobacco use, *the TBIs in this scoping review were most often combined with other evidence-based intervention components*, thus functioning in a supporting role rather than as stand-alone interventions. Of the studies assessing the utility of a TBI (eg, SMS TM) combined with evidence-based treatment, *the combinations of TBI and NRT or TBI, NRT, and cessation counseling* were most commonly associated with statistically significant differences relative to comparators in terms of BVSC or SRSC. Tobacco use pharmacotherapy combined with behavioral intervention is the recommended standard of care [87-89] and is considered a critical component of any quit attempt [90], including for individuals who identify as African American/Black, Hispanic/Latina/o, American Indian/Alaska Native [91,92]. However, pharmacotherapy for tobacco use is underused among members of these groups due to varying attitudes and beliefs about these therapies. For example, people who identify as African American/Black have lower confidence in their effectiveness relative to White people who smoke [93]; people who identify as Hispanic/Latina/o are more likely to distrust or reject medications due to a general avoidance of medications [94] coupled with a belief that willpower alone is sufficient to quit smoking [95]; and people who identify as American Indian/Alaska Native express apprehension about pharmacotherapy side effects and effectiveness [96], as well as distrust related to racism [97]. Two (8%) of the 26 included studies [26,51] mentioned disparities in the use of pharmacotherapy for members of these groups, and 1 (4%) [41] discussed at greater length the negative attitudes about NRT among people who identify as American Indian and the inconsistent availability of NRT on or near American Indian reservations. If TBIs are largely intended to support other evidence-based tobacco use therapies, it is important that *greater attention is given to existing ethnocultural concerns surrounding pharmacotherapies such as NRT* [1]. TBIs that are meant to be combined with pharmacotherapies should address these ethnocultural concerns and, when appropriate, emphasize the safety and efficacy of pharmacotherapies and attempt to dispel myths (ie, people who use NRT lack willpower) that may perpetuate the community’s negative perception of them. It is notable, however, that 2 stand-alone TBIs, including computer-delivered 5As [39] and SMS TM intervention by Mason et al [30,31], were found efficacious (BVSC at 10 weeks [39] and self-reported smoking reduction at 6 months [30,31]) compared with controls. These TBIs warrant closer attention, given their potential to impact tobacco use independently.

### Race and Ethnicity Consciousness

In her book *Race After Technology* [98], Benjamin poses the question “Can we develop a race-conscious orientation to emerging technology, not only as a mode of critique but as a prerequisite for designing technology differently?” This challenge provided direction for the race and ethnicity consciousness analysis. Our intention was to highlight areas for improvement, as well as the work of researchers whose efforts provide insights into how TBIs for tobacco use have been or may be designed to promote health equity. By reviewing the netted studies that met the *necessary* health equity–promoting condition of *including* underrepresented racial and ethnic group members, we sought to explore the extent to which these studies included consideration of and were explicit about at least one of the aforementioned underrepresented racial or ethnic groups in one or more manuscript sections (ie, title, introduction, method, results, and discussion; Textbox 1). Our finding that slightly more than half of these published manuscripts (15/26, 58%) were race or ethnicity conscious in at least one of the manuscript sections is a promising sign that researchers in this field are beginning to consider the influence of race and ethnicity on TBIs for tobacco use treatment, in addition to their inclusion of members of underrepresented groups.

The predicted growth of both Hispanic/Latina/o and African American/Black population groups, as well as the predicted shrinkage of the non–Hispanic/White population [99], increases the urgency for research that addresses health disparities through best practices for researching diverse groups [100]. Given the limited inclusion of members of underrepresented populations in biomedical research [11], there is a critical learning opportunity when a study does enroll a sample with racial and ethnic heterogeneity. Most studies (15/26, 58%) in this review allocated some manuscript space to acknowledging or being conscious of the fact that race or ethnicity might impact their findings. For example, Burlew et al [101] discuss barriers to methodologically sound health disparities research encountered at each stage of a research project from literature review through responsible dissemination to lay audiences and offer recommendations for addressing these challenges. A qualitative analysis of race-and ethnicity-conscious text segments excerpted from the studies included in this review, as well as a larger scoping review focusing on all other substance use (manuscript in preparation), is planned to explore the use of “best” evidence-based practices when conducting research with diverse groups [66,101,102] and to underscore insights that may help other researchers design studies with outcomes that promote health equity for members of underrepresented groups.

Recent meta-analyses have concluded that *evidence-based behavioral interventions adapted for a specific target group are more effective for that population than generic versions of the same interventions* [103,104]. Culturally appropriate strategies are important when addressing tobacco-related disparities among members of underrepresented groups, as tobacco use is associated with cultural norms and SES factors, such as education and poverty [105]. In the sample of articles in this review, many of the race- and ethnicity-conscious studies explored the role of cultural traditions, values, norms, and behaviors when providing a rationale for a given use, adaptation, or interpretation of an intervention for members of an underrepresented group. Mason et al [46] offered an exceptional example of exploring culture and context to provide a rationale for an approach to research and intervention. Through the ecological momentary assessment data collected during their RCT exploring the efficacy of their culturally adapted SMS TM intervention for tobacco use among adolescents who identify as African American/Black, the research group further explored the role of culture and context through secondary data analysis with attention to improved understanding of the time-varying effects of urban neighborhoods (tobacco outlet density and evaluations of perceived safety), specifically for adolescent African American/Black people who smoke. The finding that the TBI weakened the effect of tobacco outlet density on smoking for the treatment condition relative to the control highlights the potential that technology, such as ecological momentary assessment, can better interpret nuanced findings and tailor TBIs to align with real-life circumstances of members of underrepresented groups.

Avoiding the trap of “one size fits all” [106], culturally adapted TBIs may enhance the effectiveness of an intervention for people who identify as African American/Black, Hispanic/Latina/o, American Indian/Alaska Native. In their work to tailor an SMS TM intervention for tobacco use in rural American Indian/Alaska Native communities, Orr et al [34] described the results of focus groups with American Indian/Alaska Native tribal college students in Montana. Focus group members strongly disliked “text speak” (eg, “B4” for the word “before”) and strongly preferred positive, encouraging messages. These are significant observations that, if undiscovered, could have resulted in extensive resource waste to develop a TBI without impact, or worse, which could have exacerbated health disparities due to perceptions that evidence-based treatments are rarely aligned with American Indian/Alaska Native culture.

Finally, the example provided by Webb Hooper et al [51] regarding the power of exploring culture to interpret the impact of culturally sensitive interventions was instructive. Deep structural adaptations to the TBI were sensitive to the ethnocultural context and focused on topics that included African American/Black smoking statistics, physiological findings, smoking norms, pharmacotherapy concerns, religion and spirituality, family and collectivism, unique stressors, comorbid addiction, environmental influences, targeted marketing, menthol cigarettes, weight concerns, and working against the tobacco industry. Although the study did not find statistically significant differences between the tobacco cessation DVD intervention designed for a general audience and the culturally specific DVD intervention, the researchers did find that exposure to the latter led to greater perceived risks of smoking, in particular the disproportionate risks for African American/Black people compared with White people. Because perceptions of risk are posited to affect behavior change [107], this finding is promising and provides future direction as this was the first study to test the efficacy of the TBI content for its impact on people who identify as African American/Black.

Race- and ethnicity-conscious research makes members of underrepresented groups visible and audible. Tobacco researchers should consider the use of frameworks, such as the Public Health Critical Race Praxis [108], that offer new modes of inquiry and broaden the scope of research priorities to improve health equity. Strategies consistent with those proposed by Public Health Critical Race Praxis include prioritizing the perspectives of members of underrepresented groups through attention to *voice*. Many studies (10/26, 38%) included in this review engaged end users and community members in the assessment of the feasibility, acceptability, usability, and refinement of TBIs, raising the voices of these historically underrepresented groups. In a similar vein, several studies (6/26, 23%) elicited qualitative feedback, allowing for the exploration of different perspectives and a shift of investment away from White normativity [108]. Furthermore, data analytic plans that include separate analyses of racial and ethnic groups or examine race and ethnicity as moderators of outcomes to improve understanding of the social factors that support continued inequity in care also increase the visibility and raise the voices of members of these groups. In several included studies, race is treated as a covariate or controlled, effectively muting the potential impact of race. To promote health equity in research on TBIs for tobacco use treatment, it is necessary to make every effort to ensure that members of underrepresented groups are recruited and included in our samples, that their perspectives on our TBIs are elicited and valued, that whether effect sizes are smaller when compared with those for the overall study sample (and non–Hispanic/White individuals) we consider adaptations to increase effectiveness for these groups, that analyses are planned to enable meaningful interpretation of findings for these groups, and that we plan for dissemination of findings that reaches the communities most likely to benefit.

It is critical to observe that various social determinants of health, including socioeconomic factors, that is, low income and poverty, low educational attainment and literacy, and impediments to accessing health care (eg, lack of transportation, lack of insurance, and lack of trust in health care providers), all have the potential to mediate or moderate the effects of our available evidence-based treatments for tobacco use in the United States. The features of and pathways by which these societal conditions affect health can potentially be altered by evidence-based and informed action. In the case of TBIs for tobacco use in the near term, the low-hanging features and pathways include the provision of tools at no cost to the user; provision of interventions with search interfaces that have high error tolerance regarding spelling, textual information accompanied by audio narration, linear versus hierarchical navigation [74]; and provision of access to interventions without the requirement of transportation (eg, SMS TM instead of computer station at clinic or medical site). In the long term, the tobacco industry continues to profile members of underrepresented communities, especially African American/Black and American Indian/Alaska Native, through its disproportionate promotion of menthol products, the increased likelihood of tobacco advertisement displays, as well as the decreased likelihood that these communities are protected by smoke-free laws compared with areas with fewer African American/Black residents [109]. Thus, more efficacious, culturally sensitive, and competent educational initiatives focusing on children, as well as ongoing advocacy for policy changes responsive to the tobacco industry’s tactics, are simultaneously warranted.

### Limitations

There are several limitations to our methodology. First, the search terms used may have limited the findings included in our review, introducing selection bias. Relatedly, including only studies with ≥50% of underrepresented groups likely also precluded the discovery of other relevant, race- or ethnicity-conscious articles [20,64]. Only 7 (27%) of the 26 included studies followed participants for at least 6 months [33,34] (refer to Cochrane reviews), demonstrating the early-stage nature of the included research. Moreover, as many of the studies represent formative research, investigation of the potential differences for participants based on race or ethnicity proxies (unequal economic, educational, and social opportunities) is not to be expected due to small sample sizes. Furthermore, while we recognize that most of the included studies have small sample sizes and are underpowered, we believe that the findings are vital to understanding the state of the research base and emphasize the need for larger trials to remedy these shortcomings. In addition, only English language studies were included in the data set. While we recognize that this approach is common in systematic reviews, we also recognize that it can lead to biased interpretations of findings. In future research, we recommend the expansion of languages and countries included in the review, specifically Spanish and Native US languages, such as Navajo.

### Conclusions

Innovation and inequity can go hand in hand [98]. By identifying and comparing publications of TBIs for tobacco use that *include* individuals who identify as African American/Black, Hispanic/Latina/o, American Indian/Alaska Native, we are taking a first step to interrupt the perpetuation of disparities by exploring participant access, equity in outcomes, and the cultural relevance of the research [101]. By exploring the extent to which these studies are explicit about race and ethnicity and the impacts of TBIs on particular people, we further highlight research efforts to promote health equity. By shining a spotlight on this modest body of research and featuring insights related to the promotion of health equity, we hope to encourage other researchers to participate in best practices when engaging diverse groups in research.

## Appendix

Multimedia Appendix 1 MEDLINE (Ovid) databases: Ovid MEDLINE and Epub ahead of print, in-process, in-data-review, and other nonindexed citations and daily, 1946 to March 29, 2021.

Multimedia Appendix 2 Data extraction of items.

Multimedia Appendix 3 Study design, methods, and results of scoping review studies.

Multimedia Appendix 4 Technology-based interventions for tobacco use.

Multimedia Appendix 5 Study technology-based intervention access and inclusion.

Multimedia Appendix 6 Race and ethnicity consciousness of included studies.

Multimedia Appendix 7 PRISMA-ScR Checklist.

## Abbreviations

BVSC: bioverified smoking cessation
NRT: nicotine replacement therapy
PRISMA-ScR: Preferred Reporting Items For Systematic Reviews And Meta-Analyses Extension for Scoping Reviews
RCT: randomized controlled trial
SES: socioeconomic status
SRSC: self-reported smoking cessation
SUD: substance use disorder
TBI: technology-based intervention
TM: text messaging
VR: virtual reality

## Data Availability

The data sets generated during this study are available from the corresponding author upon reasonable request. One example of the search terms used in this review is provided in the Multimedia Appendix 1.
