# Supplementary material for: Technology-Based Interventions in Tobacco Use Treatment Among People Who Identify as African American/Black, Hispanic/Latina/o, and American Indian/Alaska Native: Scoping Review
Source: J Med Internet Res. 2024 Oct 10;26:e50748. doi: 10.2196/50748 (PMC11502986; doi:10.2196/50748)
Supplement: Multimedia Appendix 3 [file jmir_v26i1e50748_app3.docx]

| **Table 2. Study design, methods, and results of scoping review studies** | | | | | | |
| --- | --- | --- | --- | --- | --- | --- |
| **Study #** | **Design** | **N** | **Comparison Group(s)** | **Outcome:**  **BVSC*** | **Outcome:**  **SRSC*** | **Other** |
| **29.** Hicks et al., 2017 | RCT | 11 | **Stay Quit Coach** + (mCM, cessation counsel, NRT/Bupropion) **vs**  mCM, cessation counsel, NRT/Bupropion | 3/5 (Post-tx): 1/5 (I) (6m)  6/6 (Post-tx): 0/6 (C) (6m) | 66% (6/9 BVSC post-tx) reported cessation at 3m |  |
| **30**⧪ Shuter et al., 2014 | RCT | 138 | **Positively Smoke Free Web** + NRT **vs**  Cessation counsel, brochure, NRT | 7-day pp abstinence at 3m - **N/S** |  |  |
| **31**⧪ Prochaska et al., 2020 | RCT | 360 | **TTM Computer-assisted counsel** + (NRT, print manual) **vs**  Brochure, Quitline referral | 7-day pp abstinence at 3m, 6m – **N/S** | SRSC *and* SRSR  7-day pp abstinence  3m, 6m * |  |
| **32**⧪ Mason et al., 2015a | D/RCT | 72 | **PNC-Txt**: Personalized TM (MI) **vs**  General health habits TM |  | SRSR - * |  |
| **33**⧪ Mason et al., 2016b | RCT | 200 | N/A (Continuation of RCT/ Mason et al., 2015a) |  | SRSR  6m* (Intervention)  6m – **N/S** (Control) | Intentions not to smoke: increased * (I)  Intentions not to smoke: decreased * (C) |
| **34.** Forinash et al., 2018 | RCT | 30 | **Motivational TM** + (SOC) **vs**  SOC (pre-counsel, NRT or Bupropion, post-counsel) | 2 wks from quit date - **N/S** |  |  |
| **35**⧪ Vidrine et al., 2019 | RCT | 624 | **Tailored TM** + (NRT, phone counsel) **vs**  NRT, phone counsel | at 6 m  NRT (12%) – **N/S**  NRT + Text (12%) – **N/S** NRT + Text + Call * | past 30 days  NRT– **N/S**  NRT + Text– **N/S**  NRT + Text + Call * |  |
| **36.** Orr et al., 2019 | D/RCT | 487 | **Culturally tailored TM** + (NRT + cessation counsel) **vs**  NRT + cessation counsel |  |  |  |
| **37**⧪ Pollak et al., 2020 | RCT | 320 | **Scheduled Gradual Reduction TM and Supportive TM** + (cell phone, brochure) **vs**  Supportive TM, cell phone, brochure | 7-day pp  **N/S** | baseline to end of pregnancy (SRSR)  9 - 4 cig/day for both groups – **N/S** |  |
| **38.** Shuter et al., 2020 | RCT | 100 | **Positively Smoke Free (PSF):** Mobile cessation counsel + (brochure, NRT) **vs**  Cessation counsel, brochure, NRT | 7-day pp at 120 days - **N/S**  30-day pp at 120 days - **N/S** |  |  |
| **39.** Tagai et al., 2020 | F/A | 104 | **TxT2Commit** |  | 65% relapsed at 3m |  |
| **40.** Ruscio et al., 2016 | RCT | 44 | **Brief Mindfulness Practice (Brief-MP) vs**  Sham meditation (control) |  | SRSR at 2 wks, post-tx * |  |
| **41.** Ondersma et al., 2012 | RT | 110 | **Computer Delivered-5A’s,**  CD-5A’s + CM-Lite,  CM-Lite **vs** Time Controlled TAU | 7-day pp at 10 wks  CD-5A’s *  CM-Lite – **N/S**  CD-5A’s + CM Lite – **N/S** |  |  |
| **42.** Bernstein et al., 2016 | RT | 60 | **SmokeFreeTxt** +  (NRT, Quitline referral, Brochure) **vs**  Brochure (State Quitline Info) |  | 1m *  3m – **N/S** |  |
| **43.** Dignan et al., 2019 | RT | 254 | **mHealth AI**: Minimal **OR** Intense: 1) pre-cessation counsel, + 2) post-cessation counsel, + 3) mHealth, + 4) NRT = 15 tx conditions | Quit date to18m F/U- **N/S** | Quit date to18m F/U) - **N/S** |  |
| **44.** Bordnick et al., 2012 | F/A | 46 | **Virtual Reality Skills Training (VRST)** + (NRT) **vs** NRT | Cigarettes smoked (BVSR)  1-, 3-, and 6m post-tx * |  | Craving Reduction at 10 wks. * |
| **45.** Wilson et al., 2019 | D | 13 | **Stay Quit Coach** + (mCM, cessation counsel, NRT/Bupropion) **vs**  mCM, cessation counsel, NRT/Bupropion |  | 38% (5/13) of participants reported cessation post-tx |  |
| **46.** Krishnan et al., 2019 | F/E | 89 | **COach2Quit** app w/ iCO monitor + cessation counseling **vs**  Cessation counsel | 30-day F/U – **N/S** | 30-day F/U – **N/S** |  |
| **47.** Cartujano-Barrera et al., 2019 | F/A | 20 | **Latino Kick Buts (adapted Txt2stop)** | 7-day pp at 12 wks -  30% of participants |  |  |
| **48.** Mason et al., 2015b | S/A | 200 | N/A (S/A of Mason et al., 2016b) |  |  | Stress/Craving association:  stronger * (C) at 2m, 3m.  Craving over 6m  increased * (C)  decreased * (I) |
| **49.** Mason et al., 2016a | S/A | 197 | N/A (S/A of Mason et al., 2016b) |  |  | Tobacco Outlet Density/ Smoking association stronger * (C) 2m, 6m  Perceived safety/smoking association weaker * (I) 3m-6m |
| **50.** Mason et al., 2020 | S/A | 198 | N/A (S/A of Mason et al., 2015a) |  |  | Depression moderates tx effect * |
| **51.** Grau et al., 2017 | F/A | 25 | N/A (qualitative feedback from Bernstein et al., 2016 sample) |  |  |  |
| **52.** Wen et al., 2014 | D | 70 | N/A (qualitative feedback on **TxT2Commit**) |  |  |  |
| **53.** Woodruff et al., 2007 | RCT | 136 | **The Breathing Room** (VR) **vs**  Online surveys |  | Post-tx, 3m, 12m * |  |
| **54**⧪ Webb-Hooper et al., 2014 | RT | 140 | **Pathways to Freedom (PTF):** Culturally tailored DVD **vs** Standard DVD |  | 1m – **N/S** | Risk perception, Readiness to quit * |
| **Note: Blue =** Race conscious study: D = development study (formative work); F/A = feasibility/acceptability study; RCT = randomized controlled design; RT = randomized trial; S/A = secondary data analysis; TM = text message; SRSR = self-reported smoking reduction; SRSC = self-reported smoking cessation (abstinence); BVSR = bio-verified smoking reduction; BVSC = bio-verified smoking cessation (abstinence)/ PP = point-prevalence/ I = intervention, C = control, *=significance at p<.05, n/s = non-significant findings. / N/D = not described  ⧪ indicates that the study was sufficiently powered  This table reports on BVSC and SRSC for studies that include those measures. A few studies report on BVS/SRS Reduction (BVSR/SRSR) and in those cases the results are highlighted. For studies that do not, limited outcomes reported herein. See papers for additional outcomes of interest. | | | | | | |
