# Supplementary material for: Technology-Based Interventions in Tobacco Use Treatment Among People Who Identify as African American/Black, Hispanic/Latina/o, and American Indian/Alaska Native: Scoping Review
Source: J Med Internet Res. 2024 Oct 10;26:e50748. doi: 10.2196/50748 (PMC11502986; doi:10.2196/50748)
Supplement: Multimedia Appendix 4 [file jmir_v26i1e50748_app4.docx]

| **Table 3. Technology-based interventions for tobacco use** | | | | | |
| --- | --- | --- | --- | --- | --- |
| **Platform** | **#** | **TBI Name/ Description/Theoretical Foundation** | **Dosage** | **End-user Engagement**  **(With?)** | **Population/ Culture**  **(For?)** |
| **Apps** | 29 | **Stay Quit Coach:** Modeled after evidence-based treatment for smokers with post-traumatic stress disorder (PTSD). Designed to be integrated into ongoing psychotherapy; creates tailored quitting plans; interactive tools to help with urges to smoke; motivational messages; support contacts. / Cognitive behavioral therapy (CBT), Contingency management (CM) | Recommended use between counseling sessions and during post-treatment/ mCM for CO readings: 2 readings/ day / 7 weeks | Modeled after EBT for smokers with PTSD without end-user engagement | Guideline-concordant aid for **people who smoke with PTSD** |
|  | 45 | **Stay Quit Coach** Modeled after evidence-based treatment for smokers with post-traumatic stress disorder (PTSD). Designed to be integrated into ongoing psychotherapy; creates tailored quitting plans; interactive tools to help with urges to smoke; motivational messages; support contacts. / Cognitive behavioral therapy (CBT), Contingency management (CM) (tailored for patients with schizophrenia) | Recommended use between counseling sessions and during post-treatment/ mCM for CO readings: 2 readings/ day / 7 weeks | Qualitative feedback from end-users for intervention refinement | Guideline-concordant aid for **smokers diagnosed with schizophrenia, schizoaffective, or psychotic disorder** |
|  | 46 | **COach2Quit:** Prompts user to set quit date; reminds user to take e-CO breath test 2x’s/day. Following each breath test, the app sends personalized TM based on CO result from a predefined text message library (also includes graphical display of CO readings, instructions, and contact information for help) / Health Belief Model | e-CO breath test 2 times/day / 30 days | Qualitative feedback from end-users for intervention refinement | Not Specified |
| **Computer-delivered** | 30 | **Positively Smoke Free on the Web (PSFW)**: 8-session website (available in English and Spanish; written at 6^th^ grade level). Interactive features goals = to educate, motivate, and increase self-efficacy to quit. / Social Cognitive Theory (SCT) | 1 session/ week for 8 weeks | Unpublished end-user engagement (i.e., PLWH smokers) to distill content of the PSF group-based curriculum to individual web-based curriculum | Positively Smoke Free (PSF) curriculum targeting **PLWH who smoke** |
|  | 31⧫ | **Transtheoretical Model-Tailored (TTM) Intervention:** Brief motivationally tailored, computer-assisted counseling intervention program. / MI, CBT | Completed at baseline + 3m F/U/ Total intervention contact time, ~ 45 min. | No end-user engagement - community advisory board (CAB) encouraged use of technology for privacy and delivery to population | For **people using employment development departments (EDDs)** |
|  | 41⧫ | **Computer-Delivered 5-A’s:** Program features interactive 3-D narrator, clear and relevant graphics, and aural presentation of all content; CD-5As condition = 5As (Ask, Advise, Assess, Assist, Arrange) and—for those unwilling to set a quit goal—the 5Rs (Relevance, Risks, Rewards, Roadblocks, and Repetition). / Motivational Interviewing (MI) | 1 session | Feasibility/acceptability explored with /rated highly by similar samples of end-users, i.e., low-income women (Ondersma et al., 2005) | Software platform for **low-income, pregnant women** |
| **Text Message** | 32 | **Unnamed/Mason:** Adaptation of 20-minute, in-person intervention developed, tested, and shown to be effective with African American adolescents recruited from a Philadelphia public health clinic. Text message (TM) intervention is organized into 4 parts: (1) Rapport building, (2) Presenting tobacco use feedback, (3) Introducing social network information and presenting feedback, and (4) Summary and plans. / MI, Social-networking counseling | 6 texts/day + 3 booster TM/day/ for 5 days | Adaptation of face-to-face intervention to TM without end-user engagement | For **African American adolescents** recruited from public health clinics |
|  | 33 | **Unnamed/Mason:** Adaptation of 20-minute, in-person intervention developed, tested, and shown to be effective with African American adolescents recruited from a Philadelphia public health clinic. TM intervention is organized into 4 parts: (1) Rapport building, (2) Presenting tobacco use feedback, (3) Introducing social network information and presenting feedback, and (4) Summary and plans. / MI, Social-networking counseling | Messages occurred for 5 days (6 messages a day with an option for booster messages). Participants completed surveys at baseline, 1-, 3-, and 6-months post-intervention. | Adaptation of face-to-face intervention to TM without end-user engagement | For **African American adolescents** recruited from public health clinics |
|  | 34 | **Unnamed/Forinash:** Motivational TM focused on smoking cessation, pregnancy, and time for cessation medication refill. / MI | 1-40 min. SOC cessation visit, F/U call 3 days post quit date, then weekly/2 weeks + 13 TMs/ 28 days | No end-user engagement | TM for **pregnant women** |
|  | 35 | **Unnamed/Vidrine:** Designed to increase health knowledge, quit motivation, use of coping skills, support, and self-efficacy, TM tailored based on participant's first name and current smoking status (proactively assessed weekly by mobile phone), and on disease history, future disease concerns, and preferred coping skills. / CBT, motivational enhancement (MET) | TM daily/ 12 weeks (Frequency of TM highest (5/day) near time of quit date, w/ gradual reduction to 1/day | No end-user engagement | TM involved interventions (English and Spanish) targeted to smokers at neighborhood sites serving **racial/ethnic minority low SES people** |
|  | 36 | **Unnamed/Orr:** Smoking-cessation intervention for AI/AN adults (Montana) delivered through individually tailored TM (TM adapted from STop SmOking with Mobile Phones (STOMP) protocol to be consistent with AI/AN cultures). | 140 texts/ first 4–6 weeks; 60 texts/ weeks 7–26 | Adaptation of STOMP *with* end-user engagement | Culturally adapted TM for **AI/AN adults** |
|  | 37 | **Unnamed/Pollak:** A 160 message support TM library. Scheduled gradual reduction = reduce cigarettes smoked at baseline to 0 by the first day of the 4^th^/5^th^ week of study. Early messages focus on motivation, outcome expectations, and problem-solving; later messages focus on self-efficacy and problem-solving; late third trimester messages focus on known predictors of postpartum relapse. / SCT | Weeks 1–6, 1–2 TM/ day: Weeks 8-35, 3 TM / week. | No end-user engagement | Tech-based interventions for **pregnant women** |
|  | 38 | **Positively Smoke Free (PSF):** Mobile website offers a TM-based tobacco cessation program with smartphone features, including quit-day selection/calendar, educational/ motivational videos, and HELP button for cravings. / SCT | 8 sessions/ weekly | Qualitative feedback from end-users for intervention refinement | TM for **PLWH** |
|  | 39  **Text Message** | **TxT2Commit:** Tailored to the needs of low-income postpartum women w/ quit attempt during pregnancy, TM designed to target 5 psychosocial relapse risk factors: knowledge, beliefs, distress, decisional balance, and self-regulation. / C-SHIP, User-Centered Design | 3 system-initiated randomly selected TM per day/ 1m | Qualitative feedback from end-users for intervention refinement | TM intervention for cessation among **urban, underserved** **postpartum women** |
|  | 42 | **SmokeFreeTXT:** A library of 128 messages that employ principles of CBT and offers tips and strategies to remain abstinent. Some messages use EMA principles (e.g., asking subjects to respond to queries about mood, craving, tobacco use). / MI | TM sent randomly (9 am - 6 pm, 7 days/week)/ 28 days: maximum = 5 TM/day, 0 –1 EMA TM/day | No end-user engagement | TM program developed by the National Cancer Institute to be studied in a clinical population (i.e., **ED)** |
|  | 43 | **mHealth AI:** Health TM created using Theory of Planned Behavior (TPB) to identify AI-specific variables that contribute to explaining smoking behavior, challenges to abstaining from tobacco use. / TPB, Transtheoretical Model (TTM) | mHealth component:  MINIMAL = 2 TM/day:  INTENSE = 4 TM / day [11 visits across 18m] | No end-user engagement | Culturally appropriate tobacco use education and support interventions for **Northern Plains American Indians** |
|  | 47 | **Latino Kick Buts:** adaptation of Txt2Stop. TM counseling intervention available in English and Spanish that allows 3 levels of interactivity: pre-scheduled, keyword triggered, counselor personalized TM (free texting/non-keyword). / SCT | Pre-scheduled standard TM (pre-quit, quit-day, post-quit, post-quit maintenance) keyword-triggered standard TM, counselor personalized TM/ 12 weeks | Adaptation of STOMP *without* end-user engagement | Culturally adapted TM for **Latinos** |
|  | 48 | **Unnamed/Mason:** Adaptation of 20-minute, in-person intervention developed, tested, and shown to be effective with African American adolescents recruited from a Philadelphia public health clinic. TM intervention is organized into 4 parts: (1) Rapport building, (2) Presenting tobacco use feedback, (3) Introducing social network information and presenting feedback, and (4) Summary and plans. / MI, Social-networking counseling | 3 Ecological Momentary Assessment (EMA) surveys/ day (Th-S)/ 12 per month/ 6 m. Surveys = ~ 60 seconds to complete | Adaptation of face-to-face intervention to TM without end-user engagement | For **African American adolescents** recruited from public health clinics |
|  | 49 | **Unnamed/Mason:** Adaptation of 20-minute, in-person intervention developed, tested, and shown to be effective with African American adolescents recruited from a Philadelphia public health clinic. TM intervention is organized into 4 parts: (1) Rapport building, (2) Presenting tobacco use feedback, (3) Introducing social network information and presenting feedback, and (4) Summary and plans. / MI, Social-networking counseling | 3 EMA surveys/ day (Th-S)/ 12 per month/ 6 m. Surveys = ~ 60 seconds to complete | Adaptation of face-to-face intervention to TM without end-user engagement | For **African American adolescents** recruited from public health clinics |
|  | 50  **Text Message** | **Unnamed/Mason:** Adaptation of 20-minute, in-person intervention developed, tested, and shown to be effective with African American adolescents recruited from a Philadelphia public health clinic. TM intervention is organized into 4 parts: (1) Rapport building, (2) Presenting tobacco use feedback, (3) Introducing social network information and presenting feedback, and (4) Summary and plans. / MI, Social-networking counseling | 3 EMA surveys/ day (Th-S)/ 12 per month/ 6 m. Surveys = ~ 60 seconds to complete | Adaptation of face-to-face intervention to TM without end-user engagement | For **African American adolescents** recruited from public health clinics |
|  | 51 | **SmokeFreeTXT:** A library of 128 messages that employ principles of CBT and offers tips and strategies to remain abstinent. Some messages use EMA principles (e.g., asking subjects to respond to queries about mood, craving, tobacco use). / MI | TM sent randomly (9 am - 6 pm, 7 days/week)/ 28 days: maximum = 5 TM/day, 0 –1 EMA TM/day | Qualitative feedback from end-users for intervention refinement | TM program developed by the National Cancer Institute to be studied in a clinical population (i.e., **ED)** |
|  | 52 | **TxT2Commit:** Tailored to the needs of low-income postpartum women w/ quit attempt during pregnancy, TM designed to target 5 psychosocial relapse risk factors: knowledge, beliefs, distress, decisional balance, and self-regulation. /  Cognitive-Social Health Information Processing (C-SHIP), User-Centered Design | N/A: Development | Qualitative feedback from end-users for intervention refinement | Evidence-based TM intervention to prevent relapse among **underserved, inner city postpartum women** |
| **Virtual Reality** | 44⧫ | **Virtual Reality Skills Training (VRST):** During a typical VR session, while the participant is immersed in the environment, the therapist uses the VR scenario to assess and teach coping skills. / CBT | 10/ 60-min. sessions/ weekly | No end-user engagement | VR for skills training in a **general cessation program.** |
|  | 53⧫ | **The Breathing Room:** Virtual real-time counseling environment in which participants see each other as 3-dimensional figures (i.e., avatars) on their computer screens, and have real-time discussions with each other (benefits of peer-to-peer interaction) / MI | 7/ 45-min. VR world sessions/ weekly | Participatory research involving partnerships between researchers and community groups/ Qualitative feedback from end-users for intervention refinement | VR for cessation among **teenage smokers** |
| **DVD** | 54 | **Pathways to Freedom (PTF):** Culturally specific DVD combining knowledge regarding the history of smoking among African Americans, smoking cessation, and relapse prevention, with an ethnocultural context. / CBT | 2/ 60-min. views (latency between views = 1m) | Culturally specific adaptation *without* end-user engagement | Newly developed DVD-based cessation intervention designed for **African Americans** |
| **PDA** | 40 | **Brief MP:** Brief (lasting less than 3 weeks), daily mindfulness practice consists of 5 guided meditations (urge surfing, mindfulness of the breath, mindfulness of the body, mindfulness of thoughts, and mindfulness of emotions) pre-loaded on a PDA. | 1 meditation (20 min)/ day / 2 weeks | No end-user engagement | Mindfulness for cessation among **adult** smokers |
| ⧫ - TBI accessed at study site as opposed to remotely / mCM - mobile contingency management | | | | | |
