# Supplementary material for: Technology-Based Interventions in Tobacco Use Treatment Among People Who Identify as African American/Black, Hispanic/Latina/o, and American Indian/Alaska Native: Scoping Review
Source: J Med Internet Res. 2024 Oct 10;26:e50748. doi: 10.2196/50748 (PMC11502986; doi:10.2196/50748)
Supplement: Multimedia Appendix 5 [file jmir_v26i1e50748_app5.docx]

| **Table 4. Study technology-based intervention access and inclusion** | | | | | | | |
| --- | --- | --- | --- | --- | --- | --- | --- |
|  |  | **ACCESS** | | | | **INCLUSION** | |
|  |  | **Recruitment** | **Retention** | | |  |  |
| **Platform** | **Study #** | **Recruitment Method** | **Compensation** | **Platform**  **Availability** | **Tech Support Provided** | **Sample SES** | **Sample Race/ Ethnicity (%)** |
|  | **29** | Primary care provider referral. | Could earn up to $530 for abstinence (CM) | 1 | Y | N/D | **91% AA/B****  9% C |
| **Apps** | **45** | Outpatient psychiatric clinics, local clubhouses for people with serious mental illness (SMI), Craigslist and flier advertisements. | Could earn up to $530 for abstinence (CM) | 1 | Y | 70% SSI  39% < HS  31% HS/GED | **77% AA/B****  8% C  15% Multi-racial |
|  | **46** | Medical center/clinic, posters + participant referrals. | Up to $75 | 3 | Y | 83% Unemployed | **79% AA/B****  17% C  4% Multi-racial/ Other |
| **Computer-delivered** | **30** | Medical center/clinic recruitment. | Travel vouchers + $30 incentive/ study visit. | 3 | Y | 85% Unemployed | **60 AA/B%**, 45% H/L, 3% AI/AN  16% C |
|  | **31** | Employment development departments (EDDs) - direct approach by study staff, flyers posted in EDDs, word-of-mouth. | Up to $100 | 2 | N/D | 100% Unemployed  50% < 25K /yr. | **43% AA/B**, 8 H/L  27% N/H C  17% Multiracial  5% Other |
|  | **41** | Medical center/clinic recruitment. | Unlimited incentivization attempts for max of 5 episodes of reinforcement (gift cards worth $50), only at prenatal clinic visits (at least 1 wk. apart) | 1 | N/D | N/D | **81.8% AA/B***, ** |
| **Text Message** | **32** | Respondent-driven sampling (RDS) used at community substance use facility. Each participant could recruit up to 3 peers. | ($5) / referral enrolled | 1 | Y | N/A (adolescents) | **90.8% AA/B**  5.3% C  3.9% Other |
|  | **33** | RDS used at community substance use facility. Each participant could recruit up to 3 peers. | ($5) / referral enrolled | 1 | Y | N/A (adolescents) | **90.5% AA/B****  6.5% C  3% Other |
|  | **34** | Medical center/clinic recruitment and clinical pharmacy team referral. | N/D | 3 | N/D | 100% Public insurance | **76.4% AA/B****  20.1% C* |
|  | **35** | Churches, public housing sites, and community centers. | N/D | 1 | N/D | 55.3% <$10K/yr. 70.0% </= HS/GED | **77.9% N/H AA/B****  8.8% H/L  10.2% N/H C  3% Other |
|  | **36** | Original plan: Tribal colleges, however, low numbers forced recruitment through quitlines instead. | N/D | N/A | N/D | ~ 50% HS/GED or less | **100% AI/AN**** |
|  | **37** | Chart review to identify women w/ history of tobacco use + assessment of smoking status for all new obstetric patients. | Up to $95 | 1 | N/D | 57% HS or less  66% Unemployed  80% ‘expressed financial insecurity’ | **53% AA/B**  39% C  8% Multi-racial/Other |
|  | **38** | N/D | Travel Voucher + $30 incentive/ study visit | 3 | Y | ~ 80% Unemployed | **78.8% AA/B**, 3.8 AI/AN**  15.4% C  1.9% A/PI |
|  | **39** | Flyers and staff referrals at 3 WIC clinics. | Up to $65 for surveys | 1 | Y | 67% </= 15K/year | **63.4% N/H AA/B**  12.2% N/H C 24.4% Other |
|  | **42** | Medical center/clinic recruitment. | N/D | 3 | N/D | 78% Medicaid  64% </=HS/GED | **45% AA/B**, **21.7 H/L**  45% C  10% Other |
|  | **43**  **Text Message** | Self-identify/self-refer following promotions disseminated via radio, newspaper, and flyers at community and social events, activities sponsored by the Northern Plains Comprehensive Cancer Control Program, at Indian Health Service and Tribal and Urban Indian clinics, and at markets, casinos, tribal headquarters, and chapter houses. Subjects on Rosebud Reservation were also referred by pharmacists and healthcare providers. | Up to $35 | 1 | N/D | 78% HS/GED or greater | **100% AI/AN**** |
|  | **47** | Supermarkets, safety-net clinics, community centers, and churches | N/D | 3 | N/D | 80% HS or less  75% Uninsured | **100% H/L**** |
|  | **48** | RDS used at community substance use facility. Each participant could recruit up to 3 peers. | ($5) / referral enrolled | 1 | Y | N/A (adolescents) | **90.5% AA/B****  6.5% C  3% Other |
|  | **49** | RDS used at community substance use facility. Each participant could recruit up to 3 peers. | ($5) / referral enrolled | 1 | Y | N/A (adolescents) | **90.5% AA/B****  6.5% C  3% Other |
|  | **50** | RDS used at community substance use facility. Each participant could recruit up to 3 peers. | ($5) / referral enrolled | 1 | Y | N/A (adolescents) | **90.5% AA/B****  6.5% C  3% Other |
|  | **51** | RDS used at community substance use facility. Each participant could recruit up to 3 peers. | N/D | 3 | N/A | 76% Medicaid/ Medicare | **48% AA/B**, **12 H/L 4 AI/AN**  40% C  8% Multi-racial |
|  | **52** | Flyers and staff referrals at 3 Women, Infants, and Children (WIC) clinics. | N/A | N/A | N/A | ~ 60% < 15K/ yr.  ~ 35% 15K-30K/yr.  ~ 50% Unemployed | **~ 52% AA/B**, 20 H/L  25% C  3% Other |
|  | **44** | Television and newspaper advertisements or referred by area professionals. | N/D | 2 | N/A | N/D | **85% AA/B**, 4.5 H/L**  10.5% C |
| **Virtual Reality** | **53** | Local high school sites: traditional HS, continuation HS, and Juvenile Court and Community School systems via classroom presentations, lunch-hour sign-up tables, flyers, posters, school newspaper ads, articles, announcements, and school liaison referrals. | Up to $50 for surveys | 2 | Y | N/A (adolescents) | **51% H/L**, 5 AA/B  28% N/H C  7% A/PI  9% Other |
| **DVD** | **54** | Flyers posted throughout the community and word of mouth. | $20 | 3 | N/A | ~ 68% < 10K/ yr. | **100% AA/B**** |
| **PDA** | **40** | A community-based sample was recruited via advertisements in English on local mass transportation, newspapers, the Internet, and flyers. | $50/ initial visit, $15/ f/u sessions, $5/ completed meditations, $1/ PDA assessment. $215 max | 1 | Y | N/D | **70% AA/B**  30% C |
|  | **Blue Fill =** Race Conscious Study**;** 1 = All given device and/or Internet, 2 = No Device and/or Internet required, 3 = Eligible *only if* own / have access to phone w/ texting capability or method for viewing DVD, N/D = not described / N/A = not applicable / CM = Contingency Management / PDA = personal digital assistant / * race/ethnicity of other participants not identified/missing data; ** sample includes 75% or more of one underrepresented racial/ethnic group. | | | | | | |
