# Supplementary material for: Technology-Based Interventions in Tobacco Use Treatment Among People Who Identify as African American/Black, Hispanic/Latina/o, and American Indian/Alaska Native: Scoping Review
Source: J Med Internet Res. 2024 Oct 10;26:e50748. doi: 10.2196/50748 (PMC11502986; doi:10.2196/50748)
Supplement: Multimedia Appendix 6 [file jmir_v26i1e50748_app6.docx]

| **Table 5. Race and ethnicity consciousness of included studies** | | | | | |
| --- | --- | --- | --- | --- | --- |
| **Study #** | **Title** | **Intro** | **Methods** | **Results** | **Discussion** |
| **29** |  |  |  |  |  |
| **30** |  |  |  |  |  |
| **31** |  |  |  |  |  |
| **32** |  |  |  |  |  |
| **33** |  |  |  |  |  |
| **34** |  |  |  |  |  |
| **35** |  |  |  |  |  |
| **36** |  |  |  |  |  |
| **37** |  |  |  |  |  |
| **38** |  |  |  |  |  |
| **39** |  |  |  |  |  |
| **40** |  |  |  |  |  |
| **41** |  |  |  |  |  |
| **42** |  |  |  |  |  |
| **43** |  |  |  |  |  |
| **44** |  |  |  |  |  |
| **45** |  |  |  |  |  |
| **46** |  |  |  |  |  |
| **47** |  |  |  |  |  |
| **48** |  |  |  |  |  |
| **49** |  |  |  |  |  |
| **50** |  |  |  |  |  |
| **51** |  |  |  |  |  |
| **52** |  |  |  |  |  |
| **53** |  |  |  |  |  |
| **54** |  |  |  |  |  |
| Blue = Manuscript section is race and/or ethnicity conscious | | | | | |
